# Editor C. N. Johnson’s Pledge

**Published:** 1914-10-15

**Authors:** 


### EDITOR C. N. JOHNSON'S PLEDGE.

First I pledge myself not to continue to think ill of any man. That I shall at times and on the impulse think ill of people is only an evidence of my own frailty, and this I pledge myself to overcome in so far as I recognize it, and as soon as I recognize it.

I pledge myself to help the unfortunate to help themselves. but I shall not add to their misfortune by assuming a burden which properly belongs to them. If perchance I am stronger than others I shall not vaunt my strength by a vulgar display of paternalism over them. If I am weaker than others then shall I not cringe at their feet by permitting them to accept a responsibility which is mine.

I pledge myself to independence and self reliance except where the fact of leaning on others is for their needed development. I accept my own destiny without fear or favor.

I pledge myself to tolerance, except that I must not tolerate anything in myself which is low or mean or which I should not wish the world to see.

I pledge myself not to offend others unless by thus offending I may show them a real fault and affect a real remedy. I shall criticize no man for the sake of criticism but I shall not with-hold criticism where it will do good—the only condition being that I must first be sure that it will do good.

I pledge myself not to judge any man by external evidence not failing to remember that there is little evidence which is not external.

I pledge myself to try to do some good deed each day whereby my fellowman may be made happier, and I make this pledge realizing the exceeding great difficulty of fulfilling it owing to the rapid succession of days and the natural laxity of human nature—my own in particular.

I pledge myself to think good thoughts in so far as I can control my thinking, not forgetting that I must change my thinking frequently to keep this pledge.

I pledge myself to look on all sides of every question which may come up for my consideration, studiously avoiding the practice all too common of seeing only one side of a question—the one which is to one's own individual advantage.

I pledge myself above all things not to make myself a burden to others by magnifying my misfortunes or by constantly complaining at fate. The ills I have I shall strive to bear patiently, and seek to hide them from the world.

I pledge myself to live a clean life, not merely upright in the eyes of the law but fulfilling as nearly as I may the essence of right living as embodied in love, charity and justice.

And I make no further pledge, conscious of the fact that my natural limitations will make it sufficiently difficult to live up to these.—*Dental Review.*

---

How admirable these sentiments; wish we could join our friend in a campaign for signatures, but we haven't the courage.—*Editor Register.*

